# Association of Botanists

**Published:** 1852-07

**Authors:** 


### ASSOCIATION OF BOTANISTS.

We learn from the Cincinnati papers, that an Association of Systematic Botanists, having for its object the study and advancement of Systematic or Scientific Botany, has been recently organized in this city; President, Dr. John A. Warder; Secretary, James W. Ward, Esq. The objects of the Society are eminently practical, embracing the classification, nomenclature and normal habits of plants and the definition of their general characters, together with the history and description of the insects that feed on and destroy them. Persons willing to co-operate with the Society in any of its objects, or desiring information in regard to specimens they may have obtained, will receive attention by communicating with Mr. Ward, the General Secretary.

In this connection we beg leave to call the attention of our readers to the *Natural History Society of Louisville*, and would say to any of them who may have specimens of minerals, fossils, or animals respecting which they are desirous of obtaining information, their wishes will be attended to if they will forward them to Dr. Clapp, the President, or to Dr. L. P. Yandell, the Secretary, at the University of Louisville.

